# Methodological considerations for documenting the energy demand of dance activity: a review

**DOI:** 10.3389/fpsyg.2015.00568

**Published:** 2015-05-06

**Authors:** Sarah Beck, Emma Redding, Matthew A. Wyon

**Affiliations:** ^1^Dance Science, Trinity Laban Conservatoire of Music and DanceLondon, UK; ^2^National Institute of Dance Medicine and ScienceLondon, UK; ^3^Research Centre for Sport, Exercise and Performance, Institute for Sport, University of WolverhamptonWolverhampton, UK

**Keywords:** dance, energy demand, cardiorespiratory fitness, dance training, dance performance

## Abstract

Previous research has explored the intensity of dance class, rehearsal, and performance and attempted to document the body's physiological adaptation to these activities. Dance activity is frequently described as: complex, diverse, non-steady state, intermittent, of moderate to high intensity, and with notable differences between training and performance intensities and durations. Many limitations are noted in the methodologies of previous studies creating barriers to consensual conclusion. The present study therefore aims to examine the previous body of literature and in doing so, seeks to highlight important methodological considerations for future research in this area to strengthen our knowledge base. Four recommendations are made for future research. Firstly, research should continue to be dance genre specific, with detailed accounts of technical and stylistic elements of the movement vocabulary examined given wherever possible. Secondly, a greater breadth of performance repertoire, within and between genres, needs to be closely examined. Thirdly, a greater focus on threshold measurements is recommended due to the documented complex interplay between aerobic and anaerobic energy systems. Lastly, it is important for research to begin to combine temporal data relating to work and rest periods with real-time measurement of metabolic data in work and rest, in order to be able to quantify demand more accurately.

## Introduction

The importance of physical fitness in dance and the level of physical fitness required of dancers is a topic of much contention within both dance teaching and training settings and in dance medicine and science literature. While physiological capacity is an important aspect of dance performance, it must also be acknowledged that dance is first and foremost an art form encompassing technical and expressive aspects. Therefore, solely examining the body's physiological adaptations to training cannot infer optimal performance. As stated by Koutedakis and Jamurtas (2004), “dance performance… is a rather complex phenomenon made up of many elements that have direct and indirect effect on outcome” (p. 651–652). Therefore, it could be argued that the presence of an underlying physical fitness foundation is an important pre-requisite to successful and sustained dance performance. Previous literature has highlighted the relationship between fatigue and injury risk with regard to the considerable, and ever increasing, physical demand placed on dancers from choreography in support of this (Koutedakis and Jamurtas, 2004; Allen and Wyon, 2008), further emphasizing the role of appropriate physical preparation in dancers. In sports, training methodologies are based upon in-depth research, which seeks to understand the response of systems of energy utilization during activities and their links to performance capabilities. While dance shares several characteristics with sport there are also fundamental differences, which could make the application of non-dance specific research to dance contexts difficult. With the growth of dance science as a relatively new field of research and its increased dissemination into studio practice, there is a need for evidence to ensure the appropriate preparation of dancers within their training.

The concept of energy demand can be simplified as the energy, or oxygen, cost of completing an activity. Previous research has aimed to classify the energy demand of aspects of dance training (class and rehearsal) and performance, leading to the common description of dance activity as: complex, diverse, non-steady state, intermittent, of moderate to high intensity, and with notable differences between training and performance intensities and durations (Cohen et al., 1982a; Schantz and Astrand, 1984; Redding and Wyon, 2003; Wyon et al., 2004, 2011; Wyon, 2005; Twitchett et al., 2009a). The diversity of dance performance is particularly highlighted in relation to differences in movement vocabulary and execution both between and within dance genres. One study has noted this diversity as comparable to that of field sports such as soccer and rugby, for which extensive analyses of demand have been undertaken in the past (Wyon et al., 2011). The physiological response of the body to dance activity is noted as complicated and difficult to describe due to these characterizing features, along with a focus on technique and skill.

Only two systematic literature reviews are available in this topic area; one examining fitness components in relation to contemporary dance (Angioi et al., 2009), and one examining existing dance medicine and science literature in Dance Sport (McCabe et al., 2013). Through both of these reviews it is clear that (a) the weak methodologies of many studies do not provide a strong evidence base, (b) studies do not differentiate between levels and styles of dance adequately, and (c) inappropriate comparisons are often drawn between dance and other forms of activity; for example a generalized concept of sport. Other dance specific review articles available include an early paper on physiological aspects of dance (Kirkendall and Calabrese, 1983), a brief review examining classical ballet (Twitchett et al., 2009a), and a review of eccentric muscular contraction in dance activity (Paschalis et al., 2012). Further comment and methodological analysis articles include: examining the dancer as an athlete (Koutedakis and Jamurtas, 2004; Allen and Wyon, 2008), reviewing aerobic and anaerobic aspects of dance (Cohen, 1984), considerations for dance fitness training (Wyon, 2005; Rafferty, 2010), and a methodological review (Redding and Wyon, 2003). While these articles are useful in framing our current understanding of the physiological demands of dance; comprehensive and consensus based conclusions currently do not seem possible largely due to the lack of consistency in methodological design, protocol, and measurement instrumentation, making comparisons between different study findings difficult.

While the above mentioned articles have examined various physiological data in dance populations, to date a comprehensive literature review has not been undertaken examining data available on the intensity and energy demand of various genres of dance. The present study aims to examine the previous body of literature and in doing so, seeks to highlight important methodological considerations for future research in this area to strengthen our knowledge base.

## Methods

Pub Med/Medline, Cochrane, and SportDiscus research databases were searched between April and August 2013 with a limit to English language publications utilizing human participants only. Articles published from 1982 to August 2013 regarding the physiological demands and adaptations to dance training and performance were considered. The Medical Subject Heading (MeSH) terms “energy demand,” “physiological intensity,” “aerobic,” “anaerobic,” “movement economy,” “efficiency” were employed in combination with “dance,” “dancer,” “ballet dance,” “contemporary dance,” “modern dance.”

Articles retrieved were assessed for relevance based on title, abstract, and full-text against the pre-determined inclusion and exclusion criteria. Inclusion criteria were: (I) examining energy demand of dance or the effects of training or performance on the cardiorespiratory fitness of dancers, (II) involving participants defined as professional and/or student dancers in pre-vocational, vocational and/or University settings, (III) examining any professionally recognized genre of dance (for the purpose of this review categorized as ballet, contemporary (incl. modern), or other), (IV) investigating any aspect of dance activity (single exercise, class, training, rehearsal, performance, or competition). Exclusion criteria were: (I) editorials, review or comment articles, and conference proceedings, (II) studies measuring physical fitness variables in a one off screen, (III) studies incorporating aerobic dance (including exercise classes), (IV) studies using dance as an intervention for general population health (i.e., elderly, youth, sedentary, etc.). Finally, reference lists of accepted articles were scanned for further appropriate publications, which were subsequently sourced and included in analysis.

**Figure 1:**
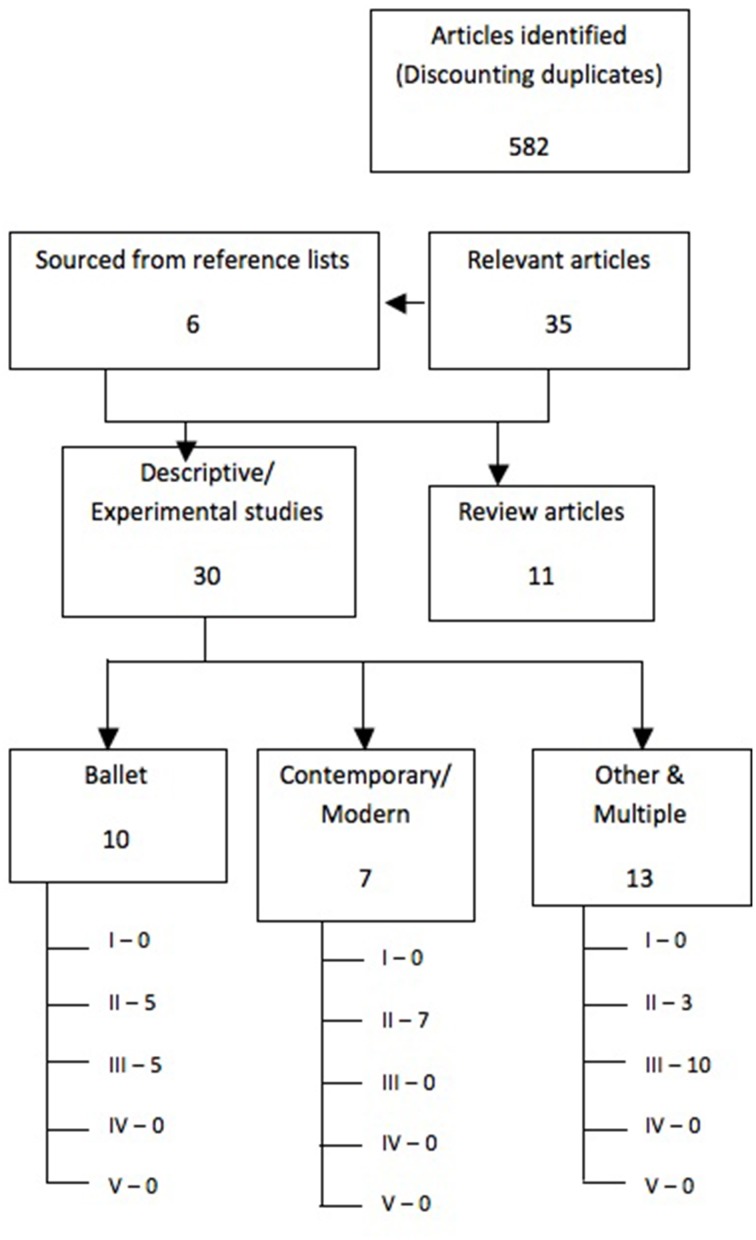
**Flow-diagram depicting the process of article identification and grading**.

An assessment of study quality of full-text articles deemed relevant was undertaken according to the grading system set out by the *ADA* evidence analysis library. The grading system, through pre-set criteria, assesses: quality, consistency, quantity, clinical impact, and generalisability. Allowing assigning of grade: I (good), II (fair), III (limited), IV (expert opinion only), or V (grade not assignable). For the purpose of this review, articles graded as expert opinion only, or grade not assignable were omitted from analysis. A flow diagram of the studies identified and included in the review appears in Figure 1. A sample review was completed by an independent reviewer to demonstrate reliability of classification.

Data analysis was conducted on accepted articles to extract relevant information for comparison and construction of evidence tables. These evidence tables form the basis of this review, allowing the summary of previous research findings and highlighting areas in need of further study.

## Results

Based on the adopted methodology and search parameters, 30 articles sourced were included in this review; 24 were descriptive studies and six involved an intervention, of which only one was clearly defined as a randomized control trial. Of the 30 articles selected, 10 articles examined ballet; seven contemporary or modern dance; nine were categorized as “other,” which included highland dance, dance sport, folk dance, tap dance, and jazz dance; and four examined multiple genres within the same study, which included ballet, contemporary, and jazz, and additionally included character dance. Terminology used to describe the genre of dance examined varied in some cases. For the purpose of this review the term “contemporary” also includes studies examining modern dance and the term “dance sport-ballroom” also includes studies examining standard or modern ballroom dance. Three studies took their sample from groups of pre-vocational, adolescent dancers, 15 examined professional dancers, 10 examined student dancers, and two looked at both student and professional dancers. Although no universal definition for categorizing an individual as a professional dancer was provided in studies examined, for the purpose of this review the term professional dancer is adopted where participants were described as such within the source study.

Quality assessment undertaken revealed the quality of evidence available to be relatively poor, with 47% graded as “limited” and the remaining 53% as “fair.” For the majority of cases, grading within the category “quantity” was the main limiting factor, with a limited number of studies confirming single study findings and low or inadequate sample sizes within studies.

Results are organized by aspects of dance activity measured in order to accurately represent different elements of a dancer's schedule. Five studies undertook measurements on more than one aspect of dance activity and therefore are included under multiple sub-headings (and appear twice or more in subsequent counts).

### Energy demand of class

Literature examining dance class generally classifies the energy demand as moderate to high and intermittent in nature; although a greater intensity and shorter duration of exercise is noted in the center phase compared to the warm-up.

Thirteen papers in total have investigated elements of dance class in various genres. Four measured only the execution of a single exercise within a class setting (**Table 2**) (Guidetti et al., 2007a, 2008; Oliveira et al., 2010; Maciejczyk and Feć, 2013), with the remaining nine examining an entire class, often drawing comparisons between different sections; most-commonly the warm-up and center phases (Table 1) (Cohen et al., 1982a; Schantz and Astrand, 1984; Rimmer et al., 1994; Dahlstrom et al., 1996; Dahlstrom, 1997; Wyon et al., 2002, 2004; Baillie et al., 2007; Guidetti et al., 2007a). Of the 13 papers examined, the majority of studies examined either ballet or contemporary dance with single, small scale studies additionally examining highland, tap, and folk dance. Groups of pre-vocational adolescents, undergraduate and graduate students, and professional dancers were examined. Data was reported on a variety of variables as displayed in Tables 1, 2. More detailed information relating to specific methodologies of each study are available in Supplementary Table 1.

**Table 1:** **Mean data of studies examining the energy demand of dance class**.

**Variable**	**References**	**Genre**	**Level**	**Gender**	**Warm-up**	**Center**	**Mean**
Mean VO_2_ (ml.kg.min^−1^)	Cohen et al., 1982a	Ballet	Pro	Female	16.49	20.06	
				Male	18.48	26.32	
	Wyon et al., 2002	Contemporary	Student	Mean	13.2	18.9	16.8
			Graduate		20.2	20.6	20.4
			Pro		15.1	21.2	18.3
	Wyon et al., 2004	Contemporary	Mixed	Female	14.67	19.39	17.42
				Male	18.65	24.78	22.06
Peak %VO_2max_	Cohen et al., 1982a	Ballet	Pro	Female		60	
				Male		71	
				Mean	51		
Mean %VO_2max_	Schantz and Astrand, 1984	Ballet	Pro	Mean	36	44.5	
Peak heart rate (b.min^−1^)	Cohen et al., 1982a	Ballet	Pro	Female		158	
				Male		178	
Median heart rate (b.min^−1^)	Dahlstrom et al., 1996	Ballet	Student	Mean	117	134	126
		Contemporary			118	137	124
		Jazz			126	153	144
		Character			133	146	140
Mean heart rate (b.min^−1^)	Cohen et al., 1982a	Ballet	Pro	Female	117	137	
				Male	134	153	
	Wyon et al., 2002	Contemporary	Student	Mean	103	125	118
			Graduate		133	132	133
			Pro		98	121	111
	Wyon et al., 2004	Contemporary	Mixed	Female	107	122	117
				Male	108	126	118
	Baillie et al., 2007	Highland	Pro	Female			151.9
	Dahlstrom, 1997	Multiple	Student	Female	132	137	134
Mean % heart rate max	Dahlstrom, 1997	Multiple	Student	Female	69	72	71
Mean blood lactate (mmol.L^−1^)	Dahlstrom et al., 1996	Ballet	Student	Mean			6.6
		Contemporary					3.8
		Jazz					2.6
		Character					4.9
	Schantz and Astrand, 1984	Ballet	Pro	Mean			3
	Dahlstrom, 1997	Multiple	Student	Female			3
Mean energy expenditure (Kcal.min^−1^)	Cohen et al., 1982a	Ballet	Pro	Female	3.96	4.86	
				Male	5.85	8.38	
	Wyon et al., 2002	Contemporary	Student	Mean	3.7	5.7	4.8
			Graduate		5.9	7.3	6.4
			Pro		4.4	6.3	5.3
Mean work time (s)	Guidetti et al., 2007a	Ballet	Student	Female			68
	Schantz and Astrand, 1984			Mean	60	25	
Mean rest time (s)	Guidetti et al., 2007a	Ballet	Student	Female			92
	Schantz and Astrand, 1984			Mean	30	80	
Mean % work time	Dahlstrom et al., 1996	Ballet	Student	Mean	60	46	55.3
		Contemporary			64	45	57.3
		Jazz			52	30	39.6
		Character			60	45	49.5
	Wyon et al., 2002	Contemporary	Student	Mean	69	33	
			Graduate		78	49	
			Pro		79	41	
	Wyon et al., 2004		Mixed	Female	74.83	41.85	
				Male	80.93	46.45	
Total time at 60–90% HR_max_ (min)	Rimmer et al., 1994	Ballet	Student	Mean			46.8

**Table 2:** **Mean data of studies examining the energy demand of a single exercise within dance class**.

**Variable**	**References**	**Genre**	**Level**	**Gender**	**Mean**
Mean VO_2_ (ml.kg.min^−1^)	Oliveira et al., 2010	Tap	Student	Female	28.2
	Maciejczyk and Feć, 2013	Folk		Female	34.23
				Male	37.75
Mean overall O_2_ cost (ml.kg^−1^)	Guidetti et al., 2007b	Ballet	Student	Female	37.5
	Guidetti et al., 2008				87.5
Mean %VO_2max_	Oliveira et al., 2010	Tap	Student	Female	68.9
	Maciejczyk and Feć, 2013	Folk		Female	81.1
				Male	74.3
Mean heart rate (b.min^−1^)	Oliveira et al., 2010	Tap	Student	Female	171
	Maciejczyk and Feć, 2013	Folk	Student	Female	178.3
				Male	167.8
Mean % heart rate max	Oliveira et al., 2010	Tap	Student	Female	83.8
	Maciejczyk and Feć, 2013	Folk		Female	91
				Male	85
Mean blood lactate (mmol.L^−1^)	Oliveira et al., 2010	Tap	Student	Female	1.7
Mean %VO_2_ at lactate threshold	Oliveira et al., 2010	Tap	Student	Female	88.2
Mean aerobic system use (ml.kg^−1^)	Guidetti et al., 2007b	Ballet	Student	Female	12
	Guidetti et al., 2008			Female	63
Mean anaerobic alactic system use (ml.kg^−1^)	Guidetti et al., 2007b	Ballet	Student	Female	19.5
	Guidetti et al., 2008			Female	18
Mean anaerobic lactic system use (ml.kg^−1^)	Guidetti et al., 2007b	Ballet	Student	Female	5.5
	Guidetti et al., 2008			Female	7
Mean energy expenditure (Kcal.min^−1^)	Maciejczyk and Feć, 2013	Folk	Student	Female	10.08
				Male	14.52
Mean energy expenditure (METS)	Oliveira et al., 2010	Tap	Student	Female	8.1

Reported work and rest temporal data suggests dance class is an intermittent form activity. Differences are noted between the work to rest ratio of the warm-up/barre phase of class and that of the center/execution phase, with the center/execution phase consisting of shorter work and longer rest periods (Schantz and Astrand, 1984; Dahlstrom et al., 1996; Wyon et al., 2002, 2004). Though methodologies and variables of focus vary, the magnitude of physiological strain is described throughout the breadth of literature as moderate to high, although this is also seen to differ between different sections of the class. Mean intensity (as represented by reported aspects of VO_2_, HR and EE) of the warm-up/barre phase is consistently reported as lower than that during the center/execution phase (Cohen et al., 1982a; Schantz and Astrand, 1984; Dahlstrom et al., 1996; Dahlstrom, 1997; Wyon et al., 2002, 2004). This difference was reported as significant for both VO_2_ and HR data in four of the studies presented (Cohen et al., 1982a; Schantz and Astrand, 1984; Wyon et al., 2002, 2004).

Three studies have compared the intensity of single exercises within a class to individual lactate/anaerobic/metabolic acidosis threshold, and described demand as working close to (Maciejczyk and Feć, 2013), often above (Guidetti et al., 2007b), 10% less than and non-significantly different to (Oliveira et al., 2010) threshold levels in Folk, Ballet, and Tap dance, respectively. Largely negligible differences in the intensity of class can be seen between dance genres, when visually comparing different studies. Only one study reported direct comparisons between classes of different dance genres and found no significant differences in the median HR-values (Dahlstrom et al., 1996). One study reported no significant differences between classes in different techniques of the same genre (Wyon et al., 2004). Lastly differences between participant characteristics such a gender and training status are reported. For example, significant differences have been reported between sexes in O_2_ requirement for the whole class, warm-up, and center phases (Wyon et al., 2004). One study noted significant differences between percentage of dance-time (work-time) and mean whole class HR-values between undergraduate, graduate and professional dancers (Wyon et al., 2002). Another study found no significant difference in overall energy requirement of a single ballet exercise between dancers ranked as high or low technical ability, however did find significant differences in energy source contribution with high technical ability dancers using significantly higher aerobic source contribution and significantly lower anaerobic lactic system contribution than the low technical ability dancers (Guidetti et al., 2008).

The data drawn from previous research examined here provides basic information regarding the intensity of dance class and therefore alludes to the demand placed upon dancers during class. However, it should be noted that data are rarely presented relative to the individual dancer's fitness levels. Significant differences are noted between different phases of class, the response of different sexes and of dancers of varying technical ability, which must be taken into account during the interpretation of data sets.

### Energy demand of rehearsal

Very few studies have presented data on the energy demands of rehearsal and high variation is reported between results of those that have. Therefore, an ambiguity regarding the demands of rehearsal prevails.

Measurements during rehearsals have been undertaken in ballet (Schantz and Astrand, 1984; Rimmer et al., 1994), contemporary (Wyon et al., 2004), and highland dance (Baillie et al., 2007), with samples taken from student and/or professional dancers. Two of the papers included in this section of the review claim to also measure performance data, however Schantz and Astrand (1984) do not differentiate between measurements that took place in final rehearsals and those conducted during performance and Wyon et al. (2004) used measurements undertaken within dress rehearsals as their performance data. Therefore, all data described above is included here as measuring the physiological demands of dance rehearsal. Data available from these studies is reported in Table 3, with more detailed information relating to specific methodologies available in Supplementary Table 2.

**Table 3:** **Mean data of studies examining the demand of dance rehearsal**.

**Variable**	**References**	**Genre**	**Level**	**Gender**	**Rehearsal**	**Dress Rehearsal**
Mean VO_2_ (ml.kg.min^−1^)	Wyon et al., 2004	Contemporary	Mixed	Female	10.17	23.34
				Male	17.19	24.85
Mean heart rate (b.min^−1^)	Wyon et al., 2004	Contemporary	Mixed	Female	108	132
				Male	112	134
	Baillie et al., 2007	Highland	Pro	Female	172.6	
End blood lactate (mmol.L^−1^)	Schantz and Astrand, 1984	Ballet	Pro	Mean	11	
Mean energy expenditure (Kcal.min^−1^)	Wyon et al., 2004	Contemporary	Mixed	Female	2.63	6.67
				Male	5.93	8.49
Total mean time at 60–90% HR_max_ (min)	Rimmer et al., 1994	Ballet	Student	Mean	45.1	

Reported mean HR-values for female participants range from 108 to 176.9 b.min^−1^ across two studies (Wyon et al., 2004; Baillie et al., 2007), with the third reporting HR-values “frequently close to max” (Schantz and Astrand, 1984), and the final study reporting that dancers spend 52% of the rehearsal time working at 60–90% HR_max_ (Rimmer et al., 1994). One study described the structure of rehearsals stating that segments of dance were performed at a high intensity level followed by a period of rest (Rimmer et al., 1994). One study reported significant differences for both mean O_2_ requirement and mean HR between rehearsal and dress rehearsal measurements, with no significant differences between sexes (Wyon et al., 2004).

### Energy demand of performance

Through literature examined the intensity of dance performance is almost unanimously described as high/heavy, with an intermittent nature, utilizing both aerobic and anaerobic energy systems.

Eleven papers have measured aspects of performance or competition, although the conditions under which measurements were taken do vary (Table 3). Five studies involved simulated competition, mimicking temporal characteristics of typical competition format, in Dance Sport (Latin American or Ballroom) (Blanksby and Reidy, 1988; Kļonova and Kļonovs, 2010; Bria et al., 2011; Massidda et al., 2011; Liiv et al., 2013). Three studies have undertaken real-time measures; one during a scheduled championship competition (Baillie et al., 2007), one during stage performance (Cohen et al., 1982b), and one including readings immediately post-performance (Galanti et al., 1993). Two studies undertook retrospective analysis from video recordings of performances (Twitchett et al., 2009b; Wyon et al., 2011), and the remaining study does not specifically outline the conditions of the performance measures (Redding et al., 2009). The majority of these studies examined professional dancers, with only one study taking its sample from student dancers. Data were reported on a variety of variables as outlined in Table 4. More detailed information relating to specific methodologies of each study are available in Supplementary Table 3.

**Table 4:** **Mean data of studies examining the demand of dance performance and/or competition**.

**Variable**	**References**	**Genre**	**Level**	**Gender**	**Mean**
Peak VO_2_ (ml.kg.min^−1^)	Liiv et al., 2013	Dance sport-ballroom	Pro	Female	43.8
				Male	50.5
Mean VO_2_ (ml.kg.min^−1^)	Blanksby and Reidy, 1988	Dance sport-ballroom		Female	34.7
				Male	42.8
		Dance sport-latin		Female	36.1
				Male	42.8
Peak %VO_2max_	Liiv et al., 2013	Dance sport-ballroom	Pro	Female	88.1
Mean %VO_2max_	Blanksby and Reidy, 1988	Dance sport-ballroom		Female	82.8
				Male	82.3
		Dance sport-Latin		Female	85.9
				Male	81.9
Bria et al., 2011	Dance sport-ballroom		Female	72.5	
				Male	75.7
		Dance sport-Latin		Female	70.8
				Male	84.2
Peak heart rate (b.min^−1^)	Cohen et al., 1982b	Ballet	Pro	Mean	178.2
Redding et al., 2009	Contemporary	Pro	Mean	187	
Mean heart rate (b.min^−1^)	Cohen et al., 1982b	Ballet	Pro	Mean	160
Redding et al., 2009	Contemporary		Mean	101	
Baillie et al., 2007	Highland		Female	195	
Kļonova and Kļonovs, 2010	Dance sport-ballroom		Female	173.05	
				Male	168.8
Blanksby and Reidy, 1988	Dance sport-Latin		Female	173	
				Male	170
				Female	177
				Male	168
Mean % heart rate max	Cohen et al., 1982b	Ballet	Pro	Mean	81.5
Galanti et al., 1993	Jazz	Student	Female	94.3	
Blanksby and Reidy, 1988	Dance sport-ballroom	Pro	Female	88	
				Male	86
		Dance sport-latin		Female	91
				Male	85
Mean end blood lactate (mmol.L^−1^)	Redding et al., 2009	Contemporary	Pro	Mean	2.45
Baillie et al., 2007	Highland		Female	6.2	
Liiv et al., 2013	Dance sport-ballroom		Female	8.7	
				Male	8.0
Bria et al., 2011	Dance sport-Latin		Female	6.91	
				Male	6.50
				Female	6.04
				Male	7.95
Mean energy expenditure (total Kcal)	Massidda et al., 2011	Dance sport-Latin	Pro	Female	159.9
				Male	251
% total time dancing	Wyon et al., 2011	Ballet	Pro	Female	62.76
				Male	61.65
		Contemporary		Female	71.28
				Male	66.56
Mean time at rest (s.min^−1^)	Wyon et al., 2011	Ballet	Pro	Female	37.22
				Male	38.5
		Contemporary		Female	18.64
				Male	20.06
Mean % total time at rest	Twitchett et al., 2009b	Ballet	Pro	Mean	64.1
Mean time at very light intensity (s.min^−1^)	Wyon et al., 2011	Ballet	Pro	Female	4.88
				Male	6.21
		Contemporary		Female	8.33
				Male	8.95
Mean time at light intensity (s.min^−1^)	Wyon et al., 2011	Ballet	Pro	Female	3.55
				Male	2.85
		Contemporary		Female	16.41
				Male	13.74
Mean time at moderate intensity (s.min^−1^)	Wyon et al., 2011	Ballet	Pro	Female	8.34
				Male	5.59
		Contemporary		Female	13.77
				Male	9.99
Mean % total time at moderate intensity	Twitchett et al., 2009b	Ballet	Pro	Mean	13
Mean time at hard intensity (s.min^−1^)	Wyon et al., 2011	Ballet	Pro	Female	7.68
				Male	4.61
		Contemporary		Female	4.28
				Male	6.59
Mean % total time at high intensity	Twitchett et al., 2009b	Ballet	Pro	Mean	11.3
Mean time at very hard intensity (s.min^−1^)	Wyon et al., 2011	Ballet	Pro	Female	2.06
				Male	3.34
		Contemporary		Female	0
				Male	0.589

The data available suggests a high mean intensity of dance performance/competition across all genres, with participants frequently reported as reaching over 80% of their HR_max_(Cohen et al., 1982b; Blanksby and Reidy, 1988; Galanti et al., 1993)and/or VO_2max_(Blanksby and Reidy, 1988; Liiv et al., 2013). However, when looking more in-depth at the data available, there seems to be no agreement regarding the amount of variation both within and between performance repertoires examined. One study found significant variations in percentage time spent working at different intensities among different positions within a classical ballet company, with soloists spending a higher percentage of total time resting than principals and principals spending a higher percentage time working at moderate intensity than soloists and artists (Twitchett et al., 2009b). The same study commented on little variation between demands of different classical repertoire examined (Twitchett et al., 2009b). Similarly, when comparing end-performance blood lactate levels in contemporary dancers, no significant difference was noted in values from four different pieces of repertoire, with all measurements noted as under 4 mmol.L^−1^ (Redding et al., 2009). Conversely, one study found significant differences in blood lactate levels between the three dance techniques performed in highland dance (*p* < 0.01) (Baillie et al., 2007). In a study comparing performance repertoire of two dance genres (ballet and contemporary/modern), significant differences were found between genres for time (s.min^−1^) spent at all subjectively categorized exercise intensities (excluding “hard”). For example, significantly more time was spent at “rest” and “very hard intensities” during ballet than contemporary performance (*p* < 0.001) (Wyon et al., 2011). Based on data presented, it is currently unclear whether any generality can be drawn in the intensity of performance between or within dance genres.

### Impact of training/performance on cardiorespiratory fitness

Data presented suggests that a program of dance activity alone is insufficient to elicit improvement in cardiorespiratory fitness, with the exception perhaps of a prolonged period of performance. This is suggested as causal of the low aerobic fitness levels often reported in dancers.

Nine studies examined adaptation of elements of cardiorespiratory fitness longitudinally over a range of training approaches. Four studies did not alter the training undertaken and simply measured at multiple time-points to see the effect of a typical training/performance period (Dahlstrom et al., 1996; Koutedakis et al., 1999; Wyon and Redding, 2005; Martyn-Stevens et al., 2012) (Table 5). However, five studies additionally involved intervention in the dancers' usual training schedule, by introducing fitness training programmes/sessions, or extra classes/rehearsals into the dancer's schedules (Galanti et al., 1993; Ramel et al., 1997; Koutedakis et al., 2007; Angioi et al., 2012; Mistiaen et al., 2012) (Table 6). More detailed information relating to specific methodologies of each study are available in Supplementary Table 4.

**Table 5:** **Mean data of studies examining measures of cardiorespiratory fitness pre-, (mid-), and post- a schedule of dance training and/or performance without intervention**.

**Variable**	**References**	**Genre**	**Level**	**Gender**	**1**	**2**	**3**
VO_2max_ (ml.kg.min^−1^)	Koutedakis et al., 1999	Ballet	Pro	Female	41.2	45.2	48.4
	Martyn-Stevens et al., 2012	Contemporary	Student		42.66	42.55	
	Dahlstrom et al., 1996	Multiple		Mean	48	49	52
Mean HR DAFT stage 5 (b.min^−1^)	Wyon and Redding, 2005	Contemporary	Pro	Mean	178.5	177.5	167
Mean %HRmax DAFT stage 5	Wyon and Redding, 2005	Contemporary	Pro	Mean	90.5	89.95	84.85
Mean BLa DAFT stage 5 (mmol.L^−1^)	Wyon and Redding, 2005	Contemporary	Pro	Mean	2.8	2.75	2.15
Mean power output (W)	Koutedakis et al., 1999	Ballet	Pro	Female	285.9	292	299
Peak power output (W)	Koutedakis et al., 1999	Ballet	Pro	Female	350	400	405
	Martyn-Stevens et al., 2012	Contemporary	Student		430.08	463.92	
Mean fatigue index (%)	Martyn-Stevens et al., 2012	Contemporary	Student	Female	33.38	38.91	

**Table 6:** **Mean data of studies examining measures of cardiorespiratory fitness pre- and post- implementation of a training intervention**.

**Variable**	**References**	**Genre**	**Level**	**Gender**	**Group**	**Pre**	**Post**
VO_2max_ (ml.kg.min^−1^)	Ramel et al., 1997	Ballet	Pro	Mean	Training	47.8	50.9
					Control	50.9	51.3
	Koutedakis et al., 2007		Student	Mean	Training	50.7	56.6
					Control	49.2	48.5
	Galanti et al., 1993	Jazz	Student	Female	37.4	43	
Mean VO_2_ at 75% HR_max_ (ml.kg.min^−1^)	Mistiaen et al., 2012	Multiple	Student	Mean	Training	27.62	29.67
Mean HR DAFT stage 5 (b.min^−1^)	Angioi et al., 2012	Contemporary	Mixed	Female	Training	196	177
					Control	196	185
Max BLa (mmol.L^−1^)	Ramel et al., 1997	Ballet	Pro	Mean	Training	9.1	9.5
					Control	9.1	8.9
Mean power output at 75% HR_max_ (W.kg^−1^)	Mistiaen et al., 2012	Multiple	Student	Mean	Training	2.28	2.44
Dance test score	Koutedakis et al., 2007	Contemporary	Student	Mean	Training	73.9	109.2
					Control	76	81.5
Aesthetic competence score	Angioi et al., 2012	Contemporary	Mixed	Female	Training	38	43
					Control	45	42

Of the four studies that did not implement an intervention to the schedules and/or training of the dancers differences can be observed in available data related to the characteristics of the dancers schedules at the time of testing, for instance between periods of rehearsal and periods of performance (Wyon and Redding, 2005). Over the course of a 3 year dance training program, with aerobic fitness measured four times per year, a significant change in predicted VO_2max_ was only observed between the first and second half of the third year of study (Dahlstrom et al., 1996). A sub-set of participants who completed measurements at all-time points recorded an mean 20% increase over the 3 years (Dahlstrom et al., 1996). Two studies examined changes in cardiorespiratory fitness during a narrower time frame of a specific rehearsal and performance cycle/season, with contrasting findings (Wyon and Redding, 2005; Martyn-Stevens et al., 2012). One study undertook a VO_2max_-test and the Wingate anaerobic bike test 10–12 weeks prior to a single performance and then again one-to-two weeks following the performance, reporting significant increases in absolute and relative peak power output and fatigue index, but no significant changes in VO_2max_-values (Martyn-Stevens et al., 2012). Whereas, significant decreases in HR-, %HR_max_-, and BLa-values at stage 5 of the DAFT were observed following an 8 week performance period (tour), indicating enhanced aerobic fitness (Wyon and Redding, 2005). In the same study, no significant differences were reported during a 12 week rehearsal period leading up to the performance period (Wyon and Redding, 2005). The fourth study measured changes in VO_2max_ and anaerobic power over the course of a 6 week rest/holiday period and over the course of the subsequent two-to-three month training period. During the rest period significant increases were reported in peak anaerobic power (*p* < 0.01) and VO_2max_ (*p* < 0.05), with a further increase reported in VO_2max_ during the subsequent training period (Koutedakis et al., 1999).

Across all nine studies, mean VO_2max_-values reported ranged from 37.4 (± 4.1) to 56.6 (± 9.3) ml.kg.min^−1^, with the top end of the range representing data recorded immediately post-aerobic training intervention. Furthermore, all five studies that implemented an intervention into the typical training schedules of the dancers, reported significant increases in measures of aerobic fitness in participants exposed to the intervention (training/exercise groups), despite differences in intervention design and length, and exercise protocol employed. One of these studies included only additional dance rehearsals, with no supplementary fitness intervention and noted improvements of 15% in a sub-maximal measure of VO_2peak_ (Galanti et al., 1993). Of the three studies that additionally undertook measurements with a control group, two also reported no significant changes in the mean VO_2max_ of control subjects (Ramel et al., 1997; Koutedakis et al., 2007), with one reporting no increase in control subjects' mean HR at DAFT stage 5 (Angioi et al., 2012). Finally, of note are the corresponding significant increases in measured aspects of dance ability/competence found only in subjects exposed to training intervention in two of the studies (Koutedakis et al., 2007; Angioi et al., 2012).

## Discussion

Evidence presented in the above review pertains to either the physiological demand of dance activity (within class, rehearsal or performance) or the longitudinal tracking of measures of aerobic and/or anaerobic fitness. Significant differences are noted in results of multiple variables presented, which are further discussed herein.

### Physiological demand of dance activity

As demonstrated by results of studies included in this review, the classification of the degree of physiological strain placed on the body during dance class involves a complex interaction of the intermittent work and rest periods and the varying intensities of work depending on specific movement vocabulary executed at different stages of the class. With most studies noting differences between two distinct phases that exist within dance classes of multiple genres, it is suggested that mean data based on the entire length of a class should be discarded. Wyon et al. (2002) recommended that classification as a high intensity intermittent activity should only be applied to the center phase work of the dance class. Furthermore, this study highlighted important influences on the total time spent dancing, such as the amount of time spent by the teachers correcting technique and the size of each group (Wyon et al., 2002).

It is evident from the review of literature that very few studies have examined dance rehearsal and amongst the data available variation is too high to allow a generalized statement of the energetic demand. In a review, Wyon (2005) noted the diversity in physical stresses previously reported and suggested the presence of a build in intensity of rehearsal closer to performance. As previously noted, a study by Wyon et al. (2004) reported significant differences for both mean O_2_ requirement and mean HR between rehearsal and dress rehearsal measurements. While the length of time between the two measurements is not stated in this instance, this nevertheless supports the suggestion that there is likely to be an influence of rehearsal status on values. It is recommended that future studies give a detailed account of the time frame in which measurements were taken (i.e., how far along the rehearsal process) and describe the temporal characteristics of the rehearsal, including time spent learning, marking, correcting, and completing full-run through's at maximum effort. Full-run through intensity is likely to closely mirror that of performance, based upon data presented by Wyon et al. (2004). Conversely, Schantz and Astrand (1984), while not differentiating full data sets, noted heart rate values 5–10 b.min^−1^ lower and blood lactate concentrations 8% lower during measurements without audience in rehearsal compared to those recorded during performance of the same dance piece in the same dancers.

While the high intensity nature of performance across dance genres is evident from data sets presented, there remains disagreement as to the diversity of different dance repertoire within single dance genres. On the one hand, there is the suggestion that performance demands vary considerably (Wyon, 2005; Baillie et al., 2007) and on the other hand, there is the suggestion that physiological demands rarely change dramatically with different repertoire (Redding et al., 2009; Twitchett et al., 2009b). Only one study has carried out direct comparison between a range of performance repertoire of different dance genres, namely ballet and contemporary, and, as previously stated, reported significant differences in time spent working at a range of exercise intensities (Wyon et al., 2011).

The main problem faced in dance training, as highlighted by the data presented, is the significant differences apparent between the energetic demand of class, rehearsal, and performance. This, along with the lack of documented positive physiological adaptation occurring through periods of dance training and rehearsal, has often lead to the conclusion that dancers are not physically prepared for performance (Wyon et al., 2004; Wyon and Redding, 2005). It is also important to acknowledge the relative nature of intensity measurements, such as those undertaken by literature presented. Wyon (2005) highlighted that while the absolute intensity of a movement sequence, be it executed as part of class, rehearsal, or performance, is set by the teacher/choreographer, the relative intensity recorded is largely influenced by the fitness of the individual dancer. It stands to reason that an individual with greater cardiorespiratory fitness would have an increased capacity to cope with the demands being placed on them during movement, therefore resulting in a documented lower relative intensity. This could be particularly important to consider in the analysis of performance data in light of suggestions that dancers do not possess the adequate fitness to cope with performance demand. Furthermore, Allen and Wyon (2008) suggest that dancers of a high skill level are likely to possess a very good economy of movement, reducing the physiological stress dancing places on them.

### Impact of training/performance on cardiorespiratory fitness

While studies have commented on the possible cardiorespiratory training effect of dance class based on total length of time spent in an appropriate training zone (suggested as 60–70% HR_max_) throughout the class, later studies have criticized this conclusion due to the small continuous time spans during which these values are attained. For example, Rimmer et al. (1994) noted that HR was not elevated into a training zone for longer than 1–6 min at any given time.

In terms of rehearsals being able to elicit positive physiological adaptation, Redding and Wyon (2003) speculated… “often rehearsal periods are short and do not allow enough time for the physiological adaptation to take place that would enable the dancer to cope with the increased demands of performance” (p. 10). Two studies have tracked change in fitness variables throughout a rehearsal period and noted no significant differences on aerobic fitness measures of VO_2max_ or HR at stage 5 of the DAFT (Wyon and Redding, 2005; Martyn-Stevens et al., 2012), but a significant difference in measures of anaerobic power (Martyn-Stevens et al., 2012). A complex interplay between time and intensity is again perhaps a limiting factor in aerobic adaptation, with the emphasis of rehearsals remaining on skill acquisition and learning.

The high intensity nature of performance has prompted speculation from research as to the possible training effect of performing, with suggestions of eliciting an aerobic training response (Blanksby and Reidy, 1988; Twitchett et al., 2009a). Intensities and durations during ballet performance have been noted as baring similarities to recommendations for interval training, with the frequency of performance instead suggested as potentially limiting factor to positive adaptation (Cohen et al., 1982b). No studies to date have clearly documented performing frequency, although one study by Wyon and Redding (2005) stated an 8 week performance period for two separate contemporary dance companies and a significant change in stage 5 DAFT HR's over this time.

Control groups of intervention studies have typically involved a sample of dancers who continue their typical training schedule, or in other words are subjected to a “dance only” program. Over a 12-week period Koutedakis et al. (2007) noted no increase in VO_2max_ under this training condition, compared to a significant increase in the sample undertaking additional supplementary fitness training. A further study included an additional contemporary dance technique class for those in the “dance only” condition and reported a non-significant change in markers of aerobic fitness (Angioi et al., 2012). Moreover, all studies included in the present review that involved supplementary fitness training intervention noted positive cardiorespiratory adaptation in exercise groups regardless of design, length, and protocol (Ramel et al., 1997; Koutedakis et al., 2007; Angioi et al., 2012; Mistiaen et al., 2012). From the literature currently available, it can subsequently be concluded that in order to elicit positive adaptation in markers of cardiorespiratory fitness, supplementary fitness training is required. Wyon and Redding (2005) further suggest that: “Cardiorespiratory training needs to be planned and managed to the same extent as the rehearsal schedule to allow the dancers to peak for each performance period as physically as they do technically” (p. 74).

### Methodological limitations

Methodological limitations of the examined literature fall under four main categories: use of mean data, prediction of VO_2_ from previous steady-state work, validity and reliability of fitness test protocols, and variables of focus.

Firstly, with all modes of dance activity described as intermittent and non-steady state in nature, the reporting of mean data is deemed somewhat invalid. As early as 1983, a review highlighted the potential underestimating of caloric expenditure in dance due to the inclusion of rest periods reducing the energy expenditure when calculated per unit of time (Kirkendall and Calabrese, 1983). Redding and Wyon (2003) also stated that “dance research that has attempted to measure work output in class, rehearsal, and performance often appears not to have considered two factors: first, that dance is not a steady-state activity… and second, that mean calculation of work output probably does not provide particularly useful information” (p. 15). However, studies have continued to report data in this way, commenting on the potential influence on results in some cases but not removing the limitation from their analyses. It is of interest to document the intensity of work periods and that of rest periods separately, as well as examine response to transitory periods, to allow an understanding not only of work intensity, but also of features of recovery as important markers of efficiency and cardiorespiratory fitness.

Secondly, the use of HR–VO_2_ relationships established during previous steady-state treadmill work to enable prediction of VO_2_ from HR's measured during dance activity, as adopted by some previous research, has been invalidated by data supplied by two studies. Schantz and Astrand (1984) found the prediction of VO_2_ in ballet class to be under-estimated by 3% (range: -9 to 21) during barre exercise, by 7% (range: -9 to 21) during moderate center work, and by 15% (−1 to 21) during severe center work. The findings of a later paper by Redding et al. (2004) further confirmed these results, reporting differences of up to 30 b.min^−1^ between the treadmill and dance protocols at any given VO_2_-value and an increase in variability with increasing intensity, up to differences of 49 b.min^−1^ at a VO_2_ of 60 ml.kg.min^−1^.

Thirdly, the validity, reliability, and sensitivity of adopted protocols for assessing cardiorespiratory fitness are questioned. For instance, the importance of weight bearing and muscle group activation has been highlighted (Redding and Wyon, 2003), suggesting that tests undertaken on a cycle ergometer, for example, may be less valid. The validity of prediction of maximal fitness from sub-maximal testing protocols is also questioned. Redding and Wyon (2003), in a review of aerobic testing methods, highlighted a necessary trade-off between reliability and validity in field predictions with potentially greater error vs. lab based controlled tests. There may also be an influence of the timing of testing, as demonstrated by significant differences in VO_2max_, before and after a 6 weeks holiday and again after a preparatory training period (Koutedakis et al., 1999). Martyn-Stevens et al. (2012) also highlighted a possible influence of familiarity with the test protocols in improvements recorded during post-testing.

The choice of variables reported largely influence the conclusions of the studies presented. Cohen et al. (1982a) highlighted the challenge of measuring absolute intensity of dance activity, due to an inability to accurately measure work output, as well as difficulties in relative measurements due to variability in the level of the dancer (time spent training/skill level) and the type and style of dance. Potential influences of muscular contraction type on metabolic data outputs have also been cited. Guidetti et al. (2007a) speculated that during a ballet class, heart rate may be more closely associated with skeletal muscle contraction than the level aerobic demand, with a potential influence of the combination of static and dynamic leg and arm movements. Paschalis et al. (2012) further cited potential influences of eccentric muscular contraction during dance movement on resting energy expenditure and movement economy. Lastly an overreliance on reporting VO_2max_ as a marker of cardiorespiratory fitness is noted. Guidetti et al. (2007a) commented on the importance of relating measurements to individual dancers ventilatory and anaerobic thresholds as these proved more sensitive to detect differences between groups of varying skill levels compared to %VO_2max_ or %HR reserve or absolute HR. Given the high intensity nature of dance, measures relating to ventilatory and/or anaerobic and/or lactate thresholds seem valid, however have been examined by very few studies to date.

In addition, it is important to account for the conditions under which data claiming to document the intensity of performance or competition is collected. One study utilizing dress rehearsal data to represent performance was previously highlighted as potentially invalid and therefore analyzed within this review as rehearsal data (Wyon et al., 2004). The use of simulated competition rather than actual competitive conditions in the available Dance Sport research (Blanksby and Reidy, 1988; Kļonova and Kļonovs, 2010; Bria et al., 2011; Massidda et al., 2011; Liiv et al., 2013) also perhaps warrants the same scrutiny. It is important to note the restriction placed on performance measurements by equipment, whereby only equipment that can be hidden under costumes is typically utilized in live performance or competition. This has lead to an over-reliance on HR data; the questionable validity and reliability of which has already been discussed. It may therefore be pertinent for future research to examine the ability of carefully designed simulated conditions such as these to represent performance and/or competition demands with an adequate degree of accuracy.

## Conclusions and future recommendations

Most individual studies examined within this review claim that dance can be classified as high intensity, intermittent exercise. Based on the temporal data examined within this review, it can be agreed that dance is an intermittent activity; however data is highlighted as inconsistent in terms of intensity, with large variability reported between study results. Furthermore, without a clearer conclusion regarding the intensity of dance training and performance, the levels of cardiorespiratory fitness required by dancers remain relatively unknown, although it has been previously suggested that “contemporary dancers need to be both aerobically and anaerobically fit in order to be prepared for the many different demands of the genre” (Redding et al., 2009, p. 8).

Previous reviews have commented on barriers to a consensual conclusion and suggest further research is necessary. Specific recommendations to guide future research, based upon the findings of this review, are as follows. Firstly, research should continue to be genre specific, with detailed accounts of technical and stylistic elements of the movement vocabulary being examined. Secondly, a greater breadth of performance repertoire, within and between genres, needs to be closely examined as a fuller understanding of demand, as well as similarities and/or differences of measurements, is necessary to determine appropriate cardiorespiratory fitness levels for dancers. Thirdly, a greater focus on threshold measurements is recommended due to the documented complex interplay between aerobic and anaerobic energy systems. Lastly, it is important for research to begin to combine temporal data relating to work and rest periods with real-time measurement of metabolic data in work and rest, in order to be able to quantify demand more accurately.

### Conflict of interest statement

The authors declare that the research was conducted in the absence of any commercial or financial relationships that could be construed as a potential conflict of interest.
