# Supplementary material for: Methodological considerations for documenting the energy demand of dance activity: a review
Source: Front Psychol. 2015 May 6;6:568. doi: 10.3389/fpsyg.2015.00568 (PMC4422004; doi:10.3389/fpsyg.2015.00568)
Supplement: Supplementary file 1 [file DataSheet1.PDF]

Supplementary Material

Energy demand of dance activity and cardiorespiratory adaptation to training and performance: a systematic review

Sarah Beck[1,3], Emma Redding[1,3*], Matthew Wyon[2,3]

[1]Dance Science, Trinity Laban Conservatoire of Music and Dance, London, UK
[2]Research Centre for Sport, Exercise and Performance, Institute for Sport, University of Wolverhampton, Walsall, UK
[3] National Institute of Dance Medicine and Science, UK

*** Correspondence:** Emma Redding, Dance Science, Trinity Laban Conservatoire of Music and Dance, Faculty of Dance, Creekside, London, SE8 3DZ, UK.
e.redding@trinitylaban.ac.uk

1. Supplementary Tables

Supplementary Table 1. Summary of methods and results of studies measuring the energy demand of dance class or the execution of a single exercise within a class setting

Author (reference)	Participants	Dance Genre	Method	Results	
**Cohen et al., 1982a**	N = 15<br>(7 men,<br>8 women)<br><br>10 measured in class<br>3 completed VO_{2max} testing<br><br>Professional (principle/ solo/ corps)	Ballet	Setting:<br>Individual classes conducted for purpose of testing (not usual class), all led by the same teacher<br><br>Measurement details:<br>VO_2 collected every 2 minutes via open circuit spirometry<br>Gross caloric expenditure derived from VO_2 measures<br>HR recorded every 30 seconds via ECG	Mean VO_2 (ml.kg.min-^{1})<br>Barre exercise:<br>16.49 ± 3.38 - female<br>18.48 ± 1.95 - male	Mean VO_2 (ml.kg.min-^{1})<br>Centre exercise:<br>20.06 ± 4.13 - female<br>26.32 ± 3.39 – male
				Peak % VO_2max<br>Barre exercise:<br>51% - average	Peak % VO_2max<br>Centre exercise:<br>60% - female<br>71% - male
				Mean HR (b.min^{-1})<br>Barre exercise:<br>117 ± 20 - female<br>134 ± 15 - male	Mean HR (b.min^{-1})<br>Centre exercise:<br>137 ± 17 - female<br>153 ± 11 - male

					Peak HR (b.min^{-1})<br>Centre exercise:<br>158 - female<br>178 - male	
				Mean EE (Kcal.min^{-1})<br>Barre exercise:<br>3.96 ± 0.96 - female<br>5.85 ± 1.04 – male	Mean EE (Kcal.min^{-1})<br>Centre exercise:<br>4.86 ± 1.08 - female<br>8.38 ± 1.54 – male	
**Schantz & Astrand, 1984**	N = 13<br>(6 male,<br>7 female)<br><br>Professional	Ballet	Setting:<br>6 different classes measured (75 minutes each)<br>Work and rest periods noted in each class<br><br>Measurement details:<br>VO_2 collected via Douglas bag and analysed in a balanced spirometer<br>Fingertip BLa samples	Mean % VO_2max<br>Barre exercise:<br>36% - average	Mean % VO_2max<br>Moderate centre exercise:<br>43% - average	Mean % VO_2max<br>Severe centre exercise:<br>46% - average
				Mean BLa (mmol.L^{-1})<br>Class:<br>3 – average		
				Mean work and rest periods (sec)<br>Barre exercise:<br>Work - 60<br>Rest - 30	Mean work and rest periods (sec)<br>Moderate centre:<br>Work - 35<br>Rest - 85	Mean work and rest periods (sec)<br>Severe centre:<br>Work - 15<br>Rest - 75
**Rimmer et al., 1994**	N = 13<br>(4 male,<br>9 female)<br><br>Students<br>(theatre dance major)	Ballet	Setting:<br>90 minute class: 45-60min barre, 5-10min adagio, 20-25min petit and grand allegro<br><br>Measurement details:<br>HR recorded every minute via wireless monitor	Total mean time at 60-90% HRmax (min)<br>46.8		
				% total time at 60-90% HRmax<br>52%		
**Guidetti et al., 2007a**	N = 39<br>(female)	Ballet	Setting:<br>71min ± 7 min class<br>One dancer at a time, same pre-	*Raw class data not reported*		

	Pre-vocational students (13 low ability, 14 intermediate, 12 high)		recorded music used each time<br><br>Measurement details:<br>Continuous HR, VE, VO_2, and VCO_2 measurement by breath-by-breath analyser<br>BLa taken after each class section and 3, 6, and 10 min following class	Mean work and rest periods (sec)<br>Work - 68<br>Rest - 92
**Guidetti et al., 2007b**	N = 12<br><br>Pre-vocational students	Ballet	Setting:<br>Single exercise of continuous movement of 25 tours piques on full point performed during 30sec period<br>Evaluated in two separate sessions: one cold and one after warm up<br><br>Measurement details:<br>Continuous VO_2 measurement via breath-by-breath analyser during exercise and 25min of sitting recovery<br>BLa at rest, warm up end, 3, 6, and 10 min into recovery<br><br>Calculations:<br>Aerobic source: amount of VO_2 above resting (VO_2ex)<br>Anaerobic alactic source: estimated from fast component of recovery after exercise (VO_2al)<br>Anaerobic lactic source: estimated from blood lactate accumulation during recovery time (VO_2la-)	Mean overall O2 cost ($ml.kg^{-1}$)<br>38 ± 3 - no warm-up<br>37 ± 3 – after warm-up<br><br>Mean aerobic energy system usage ($ml.kg^{-1}$)<br>10 ± 2 - no warm-up<br>14 ± 2 - after warm-up<br><br>Mean anaerobic alactic energy system usage ($ml.kg^{-1}$)<br>21 ± 2 - no warm-up<br>18 ± 2 - after warm-up<br><br>Mean anaerobic lactic energy system usage ($ml.kg^{-1}$)<br>7 ±1 - no warm-up<br>4 ±1 - after warm-up
**Guidetti et al., 2008**	N = 25 (female)<br><br>Pre-vocational	Ballet	Setting:<br>Single exercise lasting 210sec (72bpm, 4/4 music) of grand adage on pointe in centre (warm up of pre-	Mean overall O_2 cost ($ml.kg^{-1}$)<br>81 ± 10 - group A<br>94 ± 9 - group B

	students<br>(13 low ability,<br>12 high ability)		barre and plié at bar)<br><br>Measurement details:<br>Continuous VO_2 measurement via breath-by-breath analyser<br>BLa taken 1, 3, 5, and 7 min into recovery<br><br>Calculations:<br>Aerobic source: VO_2 above rest during the exercise,<br>Anaerobic alactic source: estimated from the fast component of the post-grand adage exercise VO_2,<br>Anaerobic lactic source – net blood lactate accumulation during recovery – subtracking rest value from peak value. Energy equivalent of 1mmol.L BLa increase assumed to be 3ml O_2 kg	Mean aerobic energy system usage ($ml.kg^{-1}$)<br>53 ± 6 – group A<br>73 ± 8 – group B		
				Mean anaerobic alactic energy system usage ($ml.kg^{-1}$)<br>19 ± 2 – group A<br>17 ± 2 – group B		
				Mean anaerobic lactic energy system usage ($ml.kg^{-1}$)<br>10 ± 1 – group A<br>4 ± 1 – group B		
**Wyon et al., 2002**	N = 27<br><br>Students<br>(10 female – final year)<br><br>Graduates<br>(3male,<br>4 female)<br><br>Professionals<br>(4male,<br>6 female)	Modern/ Contemporary	Setting:<br>All participant groups measured in situ (different classes)<br>Classes of 1.5hr duration<br><br>Measurement details:<br>VO_2 and HR data collected continuously via breath-by-breath analyser<br>Caloric expenditure was calculated from oxygen uptake data (4.8 kcal/L O_2)<br>Classes filmed in order to calculate work-to-rest ratio	Mean VO_2 ($ml.kg.min^{-1}$)<br>16.8 ± 2.3 – undergrad<br>20.4 ± 4.8 – graduates<br>18.3 ± 3.8 –professional	Mean VO_2 ($ml.kg.min^{-1}$)<br>Warm up phase:<br>13.2 ± 2.9 – undergrad<br>20.2 ± 5.5 – graduates<br>15.1 ± 3.5 -professional	Mean VO_2 ($ml.kg.min^{-1}$)<br>Centre phase:<br>18.9 ± 2.8 – undergrad<br>20.6 ± 4.7 – graduates<br>21.2 ± 4.1 -professional
				Mean HR ($b.min^{-1}$)<br>118 ± 9 – undergrad<br>133 ± 7 – graduates<br>111 ± 9 – professionals	Mean HR ($b.min^{-1}$)<br>Warm up phase:<br>103 ±7 – undergrad<br>133 ± 7 – graduates<br>98 ± 12 - professionals	Mean HR ($b.min^{-1}$)<br>Centre phase:<br>125 ± 14 – undergrad<br>132 ± 11 – graduates<br>121 ± 12 - professionals
				Mean EE ($Kcal.min^{-1}$)<br>4.8 ± 0.9 – undergrad<br>6.4 ± 2.4 – graduates<br>5.3 ± 1.6 – professionals	Mean EE ($Kcal.min^{-1}$)<br>Warm up phase:<br>3.7 ± 0.8 – undergrad<br>5.9 ± 2.3 – graduates<br>4.4 ± 1.4 - professionals	Mean EE ($Kcal.min^{-1}$)<br>Centre phase:<br>5.7 ± 1.0 – undergrad<br>7.3 ± 3.3 – graduates<br>6.3 ± 1.7 - professionals

					Mean % work time (of total time)<br>Warm up phase:<br>69 ± 4 – undergrad<br>78 ± 7 – graduates<br>79 ± 6 - professional	Mean % work time (of total time)<br>Centre phase:<br>33 ± 15 undergrad<br>49 ± 4 – graduates<br>41 ±12 - professional
**Wyon et al., 2004**	N = 40<br><br>Mixed:<br>student,<br>graduate,<br>professional	Modern/<br>Contemporary	Setting:<br>All participant groups measured in situ (different classes)<br><br>Measurement details:<br>VO_2 and HR data collected continuously via breath-by-breath analyser<br>Filmed in order to calculate work-to-rest ratio	Mean VO_2 ($ml.kg.min^{-1}$)<br>17.42 ± 2.75 – female<br>22.06 ± 5.86 – male	Mean VO_2 ($ml.kg.min^{-1}$)<br>Warm up phase:<br>14.67 ± 3.87 - female<br>18.65 ± 4.72 – male	Mean VO_2 ($ml.kg.min^{-1}$)<br>Centre phase:<br>19.39 ± 3.24 - female<br>24.78 ± 7.07 – male
				Mean HR ($b.min^{-1}$)<br>117 ± 11.58 - female<br>118 ± 15.24 - male	Mean HR ($b.min^{-1}$)<br>Warm-up phase:<br>107 ± 16.6 - female<br>108 ± 19.72 - male	Mean HR ($b.min^{-1}$)<br>Centre phase:<br>122 ± 12.58 - female<br>126 ± 14.63 - male
				Mean EE ($Kcal.min^{-1}$)<br>4.73 ± 0.81 - female<br>6.67 ± 1.95 - male	Mean EE ($Kcal.min^{-1}$)<br>Warm-up phase:<br>3.97 ± 0.82 - female<br>4.73 ± 0.81 - male	Mean EE ($Kcal.min^{-1}$)<br>Centre phase:<br>5.67 ± 1.08 - female<br>8.06 ± 2.77 - male
					Mean % work time (of total time)<br>Warm up phase:<br>74.83 ± 7.88 - female<br>80.93 ± 5.32 - male	Mean % work time (of total time)<br>Centre phase:<br>41.85 ± 13.28 - female<br>46.45 ± 10.07 - male
**Dahlstrom et al., 1996**	N = 16<br>Students	Multiple:<br>Ballet,<br>Modern, Jazz,<br>Character	Settings:<br>2 classes in each of the 4 styles measured,<br>2 students per lesson (total of 32 measurement occasions)<br><br>Measurement details:<br>HR measured continuously by ECG tape recorder with 2 electrodes<br>Fingertip blood samples obtained 3-6min after class	Me**d**ian HR ($b.min^{-1}$)<br>134 ± 17 – all<br>126 ±22 – ballet<br>124 ± 13 – modern<br>144 ± 13 – jazz<br>140 ±13 – character	Me**d**ian HR ($b.min^{-1}$)<br>Warm-up phase:<br>123 ± 18 – all<br>117 ± 25 – ballet<br>118 ± 13 – modern<br>126 ± 20 – jazz<br>133 ± 16 - character	Me**d**ian HR ($b.min^{-1}$)<br>Execution phase:<br>143 ± 15 – all<br>134 ± 25 – ballet<br>137 ± 11 – modern<br>153 ± 10 – jazz<br>146 ± 6 - character
				Mean BLa ($mmol.L^{-1}$)<br>End class:<br>6.6 ± 1.0 – ballet<br>3.8 ± 0.6 – modern<br>2.6 ± 0.8 – jazz<br>4.9 ± 1.3 – character		

				Mean % work time (of total time)<br>55.3 ± 2 – ballet<br>57.3 ± 1.9 – modern<br>39.6 ± 0.7 - jazz<br>49.5 ± 0.0 – character	Mean % work time (of total time)<br>Warm up phase:<br>60 – ballet<br>64 – modern<br>52 – jazz<br>60 - character	Mean % work time (of total time)<br>Execution phase:<br>46 – ballet<br>45 – modern<br>30 – jazz<br>45 - character
					Mean work and rest periods (sec)<br>Warm-up phase:<br>Work - 102<br>Rest - 65	Mean work and rest periods (sec)<br>Execution phase:<br>Work - 44<br>Rest - 63
**Dahlstrom, 1997**	N = 11 (female)<br><br>Students	Multiple: Ballet, Modern, Jazz, Character	Setting:<br>2 classes lasting 80min (67-87min), separated by 1-2 weeks<br><br>Measurement details:<br>HR recorded every 15 seconds using Polar HR monitor<br>Fingertip blood samples taken 1-2mins after class	Median HR (b.min^{-1})<br>134 ± 13 – class<br>132 ± 16 – warm-up phase<br>137 ± 12 – execution phase<br><br>Median % HRmax<br>71 ± 7 – class<br>69 ± 9 – warm-up phase<br>72 ± 6 – execution phase<br><br>Median BLa (mmol.L^{-1})<br>3.0 ± 0.8		
**Baillie et al., 2007**	N = 9 (female)<br><br>Professionals (championship competitors)	Other: Highland	Setting:<br>Class consisting of: 9 minute warm-up, 29 minutes of technique, 9 minutes of practice, total of 25mins of rest periods<br><br>Measurement details:<br>HR recorded every 5 seconds via telemetric HR monitor	Mean HR (b.min^{-1})<br>151.9 ± 7.4		
**Oliveira et al., 2010**	N = 8 (female)<br><br>Students	Other: Tap	Setting:<br>Single exercise of traditional American tap in 9 stages of 3 minutes with 1 minute rest between<br><br>Measurement details:	Mean VO_2 (ml.kg.min^{-1})<br>28.2 ± 4.6<br><br>Mean % VO_2max<br>68.9 ± 11.3<br><br>Mean % LT-VO_2<br>88.2 ± 15.7		

			Continuous VO_2, VCO_2, RER, and metabolic equivalent measured using a breath-by-breath analyzer,<br>BLa measured from the earlobe at each 1min interval between stages and throughout rest period<br>RPE collected throughout	Mean HR ($b.min^{-1}$)<br>171.0 ± 15.0<br>Mean % HRmax<br>83.8 ± 6.2<br>Mean % LT-HR<br>93.0 ± 10.8<br>Mean end BLa ($mmol.L^{-1}$)<br>1.7 ± 0.4<br>Mean EE (METS)<br>8.1 ± 1.3<br>Mean RPE<br>13.0 ± 2.0
**Maciejczyk & Feć, 2013**	N = 8<br>(4 female, 4 male)<br>Students	Other:<br>Folk	Setting:<br>Single exercise (Mazur) completed for 8 minutes<br><br>Measurement details:<br>VO_2 calculated by workrate-HR linear relationship and HR-O_2 linear relationship using data from prior treadmill test,<br>HR continuously recorded,<br>Energy expenditure estimated by indirect calorimetry (assumed as 5Kcal per L O_2)	Mean VO_2 ($ml.kg.min^{-1}$)<br>34.23 ± 4.36 - female<br>37.75 ± 2.05 - male<br>Mean % VO_2max<br>81.1 ± 12.23 - female<br>74.3 ± 12.47 - male<br>Mean HR ($b.min^{-1}$)<br>178.3 ± 5.62 - female<br>167.8 ± 16.68 - male<br>Mean % HRmax<br>91.0 ± 3.83 - female<br>85.0 ± 8.68 – male<br>Mean EE ($Kcal.min^{-1}$)<br>10.08 ± 2.03 – female<br>14.52 ± 2.09 - male

Supplementary Table 2. Summary of methods and results of studies measuring the energy demand of dance rehearsal

Author (reference)	Participants	Dance Genre	Method	Results	
**Schantz & Astrand, 1984**	N = 5<br>Professionals	Ballet	Setting:<br>Measurement during final rehearsals (or performance) of Giselle, Sleeping Beauty, Les Noces, and Pulcinella<br><br>Measurement details:<br>HR measured using ECG (radio telemetry)<br>Fingertip BLa samples	HR "frequently close to max"	
				Peak BLa (mmol.L^{-1})<br>11	
**Rimmer et al., 1994**	N = 13<br>(4 male,<br>9 female)<br><br>Students<br>(theatre dance major)	Ballet	Setting:<br>Series of practice sessions consisting of the dance pieces which were 1.5 to 6 minutes long<br>Rehearsals ranged from 28-156 minutes (mean 80.5min)<br><br>Measurement details:<br>HR recorded every minute via ECG	Total mean time at 60-90% HRmax (min)<br>45.1	
				% total time at 60-90% HRmax<br>56%	
**Wyon et al., 2004**	N = 40<br><br>Mixed:<br>student,<br>graduate,<br>professional	Contemporary/<br>Modern	Setting:<br>Each participant group was tested in situ<br><br>Measurement details:<br>VO_2 measured continuously via telemetric breath-by-breath analysis system<br>HR measured continuously via HR monitor<br>Filmed to calculate work: rest ratio	Mean VO_2 (ml.kg.min^{-1})<br>Rehearsal:<br>10.17 ± 6.63 – female<br>17.19 ± 3.28 – male	Mean VO_2 (ml.kg.min^{-1})<br>Dress rehearsal:<br>23.34 ± 3.83– female<br>24.85 ± 5.83 - male
				Mean HR (b.min^{-1})<br>Rehearsal:<br>108 ± 26.31 – female<br>112 ± 6.44 – male	Mean HR (b.min^{-1})<br>Dress rehearsal:<br>132 ± 9.76 – female<br>134 ± 14.28 – male
				Mean EE (kcal.min^{-1})<br>Rehearsal:<br>2.63 ± 1.87 - female<br>5.93 ± 1.33 – male	Mean EE (kcal.min^{-1})<br>Dress rehearsal:<br>6.67 ± 1.05 - female<br>8.49 ± 2.58 - male

**Baillie et al., 2007**	N = 9 (female)<br><br>Professionals (championship competitors)	Other: Highland	Setting:<br>77 minute rehearsal consisting of performance of 3 dances with 50 minutes recovery<br><br>Measurement details:<br>HR recorded every 5 seconds using telemetric HR monitors	Mean HR($b.min^{-1}$)<br>172.6 ± 5.4 – all<br>165.6 ± 6.1 – highland fling<br>176.9 ± 8.1 – sword dance<br>174.0 ± 6.0 – Sean Truibhas

Supplementary Table 3. Summary of methods and results of studies measuring the energy demand of dance performance

Author (reference)	Participants	Dance Genre	Method	Results
**Cohen et al., 1982b**	N = 13<br>(6 male,<br>7 female)<br><br>Professionals<br>(1 principle,<br>5 soloists,<br>7 corps)	Ballet	Setting:<br>Dancer's monitored one at a time through an entire act of classical ballet or contemporary rep using classical technique<br><br>Measurement details:<br>HR's continuously recorded on stage during actual performance (radio telemetry)<br>Dance action recorded and timed with a stopwatch<br>Results presented based on individual dancer movement sequence characteristics	Mean HR ($b.min^{-1}$)<br>170 – variations<br>160-180 – allegro<br>140 - adagio<br><br>Mean % HRmax<br>85 – variations<br>80-95 – allegro<br>72 - adagio<br><br>Peak HR ($b.min^{-1}$)<br>188 - variations<br>180-197 – allegro<br>158 - adagio<br><br>Work: rest ratio<br>1:3.3 – variations<br>1.16-1.19 - allegro
**Twitchett et al., 2009**	48 performances<br>(24 male,<br>24 female)<br><br>Professionals<br>(16 principals,<br>16 soloists, 16 artists)	Ballet	Setting:<br>Retrospective video analysis conducted on performance recordings<br><br>Measurement details:<br>Data recorded in each field every 30sec<br>Exercise intensity judged by qualitative description of intensity<br>Time spent at each intensity calculated in minutes and as % of whole performance	Mean % total time at rest<br>75.2 ± 15.1 – soloists<br>53 ± 24.1 - principals<br><br>Mean % total time at moderate intensity<br>9.0 ± 5.9 - artists<br>8.7 ± 9.6 – soloists<br>17.3±12.1 - principals<br><br>Mean % total time at high intensity<br>8.6 ± 8 – soloists<br>14 ± 11.5 - principals

**Wyon et al., 2011**	N = 45 contemporary (21 males, 24 females)<br><br>N = 48 classical ballet (24 male, 24 female) (16 principles, 16 soloists, 16 artists)<br><br>Professional	Multiple: Ballet & Contemporary/ Modern	Setting:<br>Retrospective movement analysis conducted with time motion and match analysis systems on performance recordings<br><br>Measurement details:<br>Data recorded in each field every 30sec<br>Exercise intensity judged by qualitative description of intensity<br>Time spent at each intensity calculated in minutes and as % of whole performance	Mean % total time dancing<br>Ballet:<br>62.76 ± 13.74 – female<br>61.65 ± 14.33 - male	Mean % total time dancing<br>Contemporary:<br>71.28 ± 18.18 – female<br>66.56 ± 12.79 - male
				Mean time spent at rest ($s.min^{-1}$)<br>Ballet:<br>37.22 ± 13.73 – female<br>38.50 ± 14.51 - male	Mean time spent at rest ($s.min^{-1}$)<br>Contemporary:<br>18.64 ± 10.78 - female<br>20.06 ± 7.69 - male
				Mean time spent at very light intensity ($s.min^{-1}$)<br>Ballet:<br>4.88 ± 3.81 – female<br>6.21 ± 4.91 - male	Mean time spent at very light intensity ($s.min^{-1}$)<br>Contemporary:<br>8.33 ± 10.95 – female<br>8.95 ± 8.11 - male
				Mean time spent at light intensity ($s.min^{-1}$)<br>Ballet:<br>3.55 ± 3.75 – female<br>2.85 ± 2.61 – male	Mean time spent at light intensity ($s.min^{-1}$)<br>Contemporary:<br>16.41 ± 10.79 – female<br>13.74 ± 12.43 – male
				Mean time spent at moderate intensity ($s.min^{-1}$)<br>Ballet:<br>8.34 ± 7.05 – female<br>5.59 ± 4.79 - male	Mean time spent at moderate intensity ($s.min^{-1}$)<br>Contemporary:<br>13.77 ± 7.19 – female<br>9.99 ± 6.0 - male
				Mean time spent at hard intensity ($s.min^{-1}$)<br>Ballet:<br>7.68 ± 6.83 – female<br>4.61 ± 3.79 - male	Mean time spent at hard intensity ($s.min^{-1}$)<br>Contemporary:<br>4.28 ± 5.53 – female<br>6.59 ± 7.27 - male
				Mean time spent at very hard intensity ($s.min^{-1}$)<br>Ballet:<br>2.06 ± 2.89 – female<br>3.34 ± 4.04 - male	Mean time spent at very hard intensity ($s.min^{-1}$)<br>Contemporary:<br>0.00 – female<br>0.589 ± 1.08 – male

**Redding et al., 2009**	N = 8<br><br>Professionals	Contemporary/ Modern	Setting:<br>Continuous measurement while performing four dance pieces of current five piece repertoire<br><br>Measurement details:<br>Continuous HR measurement via HR monitor<br>Fingertip BLa sample taken at the end of each piece	Mean HR ($b.min^{-1}$)<br>101<br><br>Peak HR ($b.min^{-1}$)<br>187<br><br>Mean end BLa ($mmol.L^{-1}$)<br>1.6 ± 0.4 – piece 1<br>3.9 ± 1.6 – piece 2<br>2.3 ± 1.0 – piece 3<br>2.0 ± 0.4 – piece 4	
**Galanti et al., 1993**	N = 8<br>(female)<br><br>Students	Other:<br>Jazz	Setting:<br>Measurement during performance consisting of 3 choreographed sequences ranging from 2-5min in length<br><br>Measurement details:<br>HR recorded after each dance during the performance	Mean % HRmax<br>94.3 ± 6.8 – all<br>93.5 ± 11.9 - Dance 1<br>100.0 ± 4.1 - Dance 2<br>89.6 ± 7.2 - Dance 3	
**Baillie et al., 2007**	N = 9<br>(female)<br><br>Professionals (championship competitors)	Other:<br>Highland	Setting:<br>3 dances performed by each participant during one competition, with a total recovery period averaging 50minutes<br><br>Measurement details:<br>HR recorded every 5 seconds using telemetric HR monitors<br>BLa samples taken before and after each dance during competition	Mean HR($b.min^{-1}$)<br>195.0 ± 6.5 – all<br>194.2 ± 10.0 – highland fling<br>196.3 ± 6.3 – sword dance<br>194.6 ± 6.3 – Sean Truibhas<br><br>Mean BLa ($mmol.L^{-1}$)<br>End dance:<br>4.5 ± 1.86 - highland fling<br>6.9 ± 2.96 - sword dance<br>7.3 ± 2.96 - Sean Truibhas	
**Blanksby & Reidy, 1988**	N = 20<br>(10 couples:<br>10 male,<br>10 female)<br><br>Professionals	Other:<br>Dance Sport (Modern and Latin American)	Setting:<br>Simulated competition with costumes<br>Each completed modern (modern waltz, tango, foxtrot, quickstep, & Viennese waltz) and Latin American (samba, rumba, paso double, cha cha, & jive) sequence with 15-20sec	Mean VO_2 ($ml.kg.min^{-1}$)<br>Modern:<br>34.7 ± 3.8 - female<br>42.8 ± 5.7 - male<br><br>Mean % VO_2max<br>Modern:<br>82.8 ± 6.9 - female<br>82.3 ± 8 – male	Mean VO_2 ($ml.kg.min^{-1}$)<br>Latin American:<br>36.1 ± 4.1 - female<br>42.8 ± 6.9 - male<br><br>Mean % VO_2max<br>Latin American:<br>85.9 ± 4.0 - female<br>81.9 ± 2.3 - male

			break between each dance and 30mins rest between sequences<br><br>Measurement details:<br>HR recorded every 5 seconds using telemetric HR monitors<br>VO_2 values predicted from VO_2-HR relationship during previous VO_2max test	Mean HR (b.min^{-1})<br>Modern:<br>173 - female<br>170 – male	Mean HR (b.min^{-1})<br>Latin American:<br>177 – female<br>168 - male	
				Mean % HRmax<br>Modern:<br>88 ± 6 - female<br>86 ± 5 - male	Mean % HRmax<br>Latin American:<br>91 ± 6 - female<br>85 ± 7 – male	
**Kļonova & Kļonovs, 2010**	N = 24<br>(12 couples:<br>12 male,<br>12 female)<br><br>Professionals	Other:<br>Dance Sport<br>(Ballroom)	Setting:<br>Sequence completed with 15-20sec rest between dances: slow waltz, tango, Viennese waltz, slow foxtrot, quickstep<br>Completed twice: with and without partners' close contact (30min rest between)<br><br>Measurement details:<br>HR measured using HR monitors	Mean HR (b.min^{-1})<br>With contact:<br>171.3 ± 5.22 – female<br>167.2 ± 4.68 - male	Mean HR (b.min^{-1})<br>Without contact:<br>174.8 ± 5.54 – female<br>170.4 ± 5.32 - male	
**Bria et al., 2011**	N = 24<br>(12 couples, 12 male, 12 female)<br><br>Professional (6 Latin-American couples,<br>6 Standard couples)	Other:<br>Dance Sport<br>(Standard & Latin American)	Setting:<br>Simulated competition of Standard and Latin American sequences<br><br>Measurement details:<br>Continuous measurement of VO_2<br>BLa measured between dances	Mean %VO_2peak<br>Standard:<br>72.5 ± 12.8 - female<br>75.7 ± 10.6 - male	Mean %VO_2peak<br>Latin American:<br>70.8 ± 13.8 - female<br>84.2 ± 11.2 – male	
				Peak BLa<br>Standard:<br>6.91± 2.6 – female<br>6.50 ± 2.1 - male	Peak BLa<br>Latin American:<br>6.04 ± 2.5 – female<br>7.95 ± 2.1 - male	
**Massidda et al., 2011**	N = 10<br>(5 couples, 5 males and 5 females)<br><br>Professional	Other:<br>Dance Sport<br>(Latin American)	Setting:<br>Performed competitive dance simulation; semifinal and final phases each consisting of five dance types, with 30.1min rest between<br>Total length of simulation 1 hr 15 min	Mean total EE (Kcal)<br>159.9 ± 16.4 – female<br>251 ± 27.8 - male	Mean total EE (Kcal)<br>Semi-final:<br>70.2 ± 7.4 - female<br>107.7 ± 5.5 - male	Mean total EE (Kcal)<br>Final:<br>39.1 ± 5.3- *female*<br>52.8 ± 12.4 - *male*

			Measurement details:<br>Sensewear Pro armband recorded minute-by-minute analysis of Kcal and METs expenditure	
**Liiv et al., 2013**	N = 16<br>(8 couples, 8male, 8 female)<br><br>Professional (mean world ranking 1.657WDSF)	Other:<br>Dance Sport (Ballroom - Modern/ Standard)	Setting:<br>Simulated ballroom competition one trial of 11mins, 20sec recovery interval between each dance<br><br>Measurement details:<br>VO2 measurement during trial via telemetric gas analysis system<br>BLa measured at rest and 3-5mins after simulated competition	Peak VO_2 ($ml.kg.min^{-1}$)<br>43.8 ± 9.9 - female<br>50.5 ± 7.3 - male<br>Peak % VO_2max<br>88.1 – female<br>75.8 - male<br>Mean end BLa ($mmol.L^{-1}$)<br>8.7 ± 0.35 – female<br>8.0 ± 2.7 - male

Supplementary Table 4. Summary of methods and results of studies examining the impact of dance training/ performance on cardiorespiratory fitness

Author (reference)	Participants	Dance Genre	Method	Results	
**Ramel et al.,1997**	N = 20<br>(10 in control, 10 training)<br><br>Professionals	Ballet	Testing protocols:<br>VO_{2max} test: cycle ergometer to exhaustion<br>Expired gas collected continuously in a mixing chamber, measurements obtained every 20sec during exercise, VO_{2max} taken as highest value recorded during last minute of exercise,<br>Finger tip BLa samples after 4min of exercise, every 2mins, at end of test, and 4min into recovery<br><br>Intervention:<br>At least 30mins 2 times per week using aerobic training activity of their choice at 70-80% HRreserve	Mean VO_2max (ml.kg.min^{-1})<br>Pre:<br>47.8 (33.9-58.8) – training<br>50.9 (45.2-55.1) - control<br><br>Mean Max BLa (mmol.L^{-1})<br>Pre:<br>9.1 (6.1-12.9) – training<br>9.1 (4.1-13.5) - control	Mean VO_2max (ml.kg.min^{-1})<br>Post:<br>50.9 (47.7-63.5) – training<br>51.3 (40.6-55.5) – control<br><br>Mean Max BLa (mmol.L^{-1})<br>Post:<br>9.5 (6.2-13.6) - training<br>8.9 (5.9-10.1) - control
**Koutedakis et al., 1999**	N = 17<br>(female)<br><br>Professionals	Ballet	Testing protocols:<br>Treadmill ergometry: 5min warm up 9km/h, progressive 1min increments of 0.5km/h until exhaustion, VO_{2max} and VE_{max} calculated via automated gas analyser measuring respiratory parameters every 30sec<br>Anaerobic wingate test: 20sec protocol, mean power & peak power calculated<br><br>Setting:<br>Assessed just before and after summer break, during which little or no physical work was undertaken<br>8 dancers assessed a 3rd time 2-3months into new season	Mean VO2max (ml.kg.min-1)<br>41.2 ± 8.5 – pre-rest<br>45.2 ± 7.1 – post-rest<br>48.4 ± 6.8 – post-prep period<br><br>Mean average power output (W)<br>285.9 ± 41.1 – pre-rest<br>292 ± 38.5 – post-rest<br>299 ± 28.3 – post-prep period	 <br><br><br><br>Mean peak power output (W)<br>350 ± 46.2- pre-rest<br>400 ± 35.1 – post-rest<br>405 ± 33.3 – post-prep period

**Wyon & Redding, 2005**	N = 17<br>(8 males,<br>9 females)<br><br>Professionals<br>2 company's<br>(group 1/ group 2)	Contemporary/ Modern	Testing protocols:<br>Dance Aerobic Fitness Test (DAFT) completed with HR monitors worn to provide end-stage HR values<br>BLa sample 1min after testing<br><br>Setting:<br>Initial test 1-2 weeks after return from break and 2-3 weeks before start of rehearsals,<br>Second test 1 week before start of performing (week 12),<br>Final test 1 week following the end of tour (week 20)	Mean HR DAFT stage 5 (b.min^{-1})<br>Pre-rehearsal period: 167 ± 10.65 –group 1<br>190 ± 3.07 – group 2	Mean HR DAFT stage 5 (b.min^{-1})<br>Pre-performance period:<br>166 ± 10.55– group 1<br>189 ± 4.19 – group 2	Mean HR DAFT stage 5 (b.min^{-1})<br>Post-performance period:<br>155 ± 12.86 –group 1<br>179 ± 4.76 – group 2
				Mean % HRmax DAFT stage 5<br>Pre-rehearsal period:<br>84.7 ± 4.89- group 1<br>96.3 ± 1.96 – group 2	Mean % HRmax DAFT stage 5<br>Pre-performance period:<br>84.1 ± 4.99 – group 1<br>95.8 ± 2.23 – group 2	Mean % HRmax DAFT stage 5<br>Post-performance period:<br>77.9 ± 6.18 – group 1<br>91.8 ± 3.17 – group 2
				Mean end BLa DAFT stage 5 (mmol.L^{-1})<br>Pre-rehearsal period:<br>2.2 ± 0.92 – group 1<br>3.4 ± 1.12 - group 2	Mean end BLa DAFT stage 5 (mmol.L^{-1})<br>Pre-performance period:<br>2.1 ± 0.90 – group 1<br>3.4 ± 1.15 - group 2	Mean end BLa DAFT stage 5 (mmol.L^{-1})<br>Post-performance period:<br>1.5 ± 0.77 – group 1<br>2.8 ± 1.09 - group 2
**Koutedakis et al., 2007**	N = 32<br>(5 males,<br>27 females)<br>Students	Contemporary/ Modern	Testing protocols:<br>Treadmill VO_{2max}: 5 min warm up at (9km.h), followed by 0.5km.h increase in 1min increments until exhaustion<br>Dance test: choreographed movement phrase repeated until fatigue related technical detriment, marking criteria with 5 elements (posture/ alignment, use of articulation in upper body, lower body, total body coordination, presentation of movement)	Mean VO_2max (ml.kg.min^{-1})<br>Pre:<br>50.7 ± 7.5 – exercise<br>49.2 ± 5.5 - control	Mean VO_2max (ml.kg.min^{-1})<br>Post:<br>56.6 ± 9.3 – exercise<br>48.5 ± 5.4 – control	

			Intervention:<br>Aerobic training 20-40mins of swimming/ jogging/ cycling 2-3 times per week for 12 weeks, 70-75% age-predicted HR_{max}	Mean dance test score (points)<br>Pre:<br>73.9 ±16.2 – exercise<br>76.0 ± 19.4 - control	Mean dance test score (points)<br>Post:<br>109.2 ± 21.3 – exercise<br>81.5 ± 11.8 - control
**Angioi et al., 2012**	N = 24<br>(female)<br><br>14 Students,<br>10 professional	Contemporary/ Modern	Testing protocols:<br>Aerobic fitness via Dance Aerobic Fitness Test (DAFT), HR monitors worn throughout to calculate mean HR of the last minute of stage 5<br>Aesthetic Competence test consisting of a 90sec choreographed contemp dance routine scored on a scale<br><br>Intervention:<br>6 week programme<br>Conditioning group 2x1hr exercise per week (Dance specific circuit training/ whole body vibration training),<br>Control group 1 extra hour of contemporary technique class per week	Mean HR DAFT stage 5 ($b.min^{-1}$)<br>Pre:<br>196 ± 9.71 – conditioning<br>196 ± 3.59 - control	Mean HR DAFT stage 5 ($b.min^{-1}$)<br>Post:<br>177 ± 15.5 – conditioning<br>185 ± 7.07 – control
				Aesthetic competence score (points)<br>Pre:<br>38 ± 12.92 – conditioning<br>45 ± 6.22 – control	Aesthetic competence score (points)<br>Post:<br>43 ± 6.34 – conditioning<br>42 ± 3.34 - control
**Martyn-Stevens et al., 2012**	N = 18<br>(female)<br><br>Students	Contemporary/ Modern	Testing protocols:<br>VO_{2max} graded treadmill test (Bruce protocol) using a metabolic cart<br>Wingate anaerobic bike test, 30 second protocol<br><br>Setting:<br>Measured pre- and post-season over 2 days of screening; pre- 10-12 weeks before dance performance, post- 1-2 weeks after performance	Mean VO_2max ($ml.kg.min^{-1}$)<br>42.66 ± 4.33 – pre<br>42.55 ± 4.26 - post	
				Mean peak power output (W)<br>430.08 ± 61.24 – pre<br>463.92 ± 58.95 - post	
				Mean peak power output ($W.kg^{-1}$)<br>7.43 ± 1.01 – pre<br>8.00 ± 0.78 – post	
				Mean fatigue index (%)<br>33.38 ± 9.72 – pre<br>38.91 ± 7.49 - post	
**Galanti et al., 1993**	N = 8<br>(female)<br><br>Students	Other:<br>Jazz	Testing protocols:<br>VO_{2peak} graded treadmill test (Bruce protocol) to exhaustion<br>VO_2 and HR recorded using metabolic cart every 15se	Mean VO_2max ($ml.kg.min^{-1}$)<br>37.4 ± 4.1 – pre<br>43.0 ± 3.5 – post	

			Intervention:<br>Participated in dance training 4 days a week for ~90min per session, finishing with a final performance			
**Dahlstrom et al., 1996**	N = 53 *Year 1*<br>(N = 52 *Year 2*)<br>(N = 43 *Year 3*)<br><br>Students	Multiple:<br>Ballet, Modern, Jazz, Character	Testing protocols:<br>Predicted VO_{2max} from submax. cycle ergometer test 4 times per year (mid and end semester; *TP1*, *TP2*, *TP3*, *TP4*) over the 3 year program<br><br>Setting:<br>Three year undergraduate dance program (no intervention in training)	Mean VO_2 max ($ml.kg.min^{-1}$)<br>Year 1:<br>46 ± 9 – TP1<br>47 ± 8 – TP2<br>47 ± 8 – TP3<br>48 ± 9 – TP4	Mean VO_2 max ($ml.kg.min^{-1}$)<br>Year 2:<br>48 ± 9 – TP1<br>48 ± 9 – TP2<br>48 ± 8 – TP3<br>49 ± 9 – TP4	Mean VO_2 max ($ml.kg.min^{-1}$)<br>Year 3:<br>49 ± 9 – TP1<br>47 ± 9 – TP2<br>50 ± 9 – TP3<br>52 ± 10 – TP4
**Mistiaen et al., 2012**	N = 40<br>(2 male, 38 female)<br><br>Students	Multiple	Testing protocols:<br>Submax. cycle-ergometer test consisting of 25W increases until 75% of predicted HRmax VO_2 and HR monitored by breath-by-breath analysis system<br><br>Intervention:<br>6 month training programme<br>3 sessions per week of 90mins each	Mean VO_2 at 75% HRmax ($ml.kg.min^{-1}$)<br>27.62 ± 6.71 – pre<br>29.67 ± 6.10 – post<br><br>Mean power output at 75% HRmax ($W.kg^{-1}$)<br>2.28 ± 0.60 – pre<br>2.44 ± 0.60 - post		

References

Angioi, M., Metsios, G., Twitchett, E. A., Koutedakis, Y., Wyon, M. (2012). Effects of supplemental training on fitness and aesthetic competence parameters in contemporary dance: a randomised controlled trial. *Med. Probl. Perform. Ar.* 27:1, 3-8.

Baillie, Y., Wyon, M., Head, A. (2007). Highland Dance: Heart-Rate and Blood Lactate Differences Between Competition and Class. *Int. J. Sports Physiol. Perform.* 2:4, 371-376.

Blanksby, B. A., Reidy, P. W. (1988). Heart rate and estimated energy expenditure during ballroom dancing. *Brit. J. Sport Med.* 22:2, 57-60. doi:10.1136/bjsm.22.2.57

Bria, S., Bianco, M., Galvani, C., Palmieri, V., Zeppilli, P., Faina, M. (2011). Physiological characteristics of elite sport-dancers. *J. Sport Med. Phys. Fit.* 51:2, 194-203.

Cohen, J. L., Segal, K. R., McArdle, W.D. (1982b). Heart rate response to ballet stage performance. *Physician Sportsmed.* 10:11, 120-122;125-130;133.

Cohen, J. L., Segal, K. R., Witriol, I., McArdle, W. D. (1982a). Cardiorespiratory responses to ballet exercise and the VO2max of elite ballet dancers. *Med. Sci. Sport. Exerc.* 14:3, 212-217.

Dahlstrom, M. (1997). Physical effort during dance training: a comparison between teachers and students. *J. Dance Med. Sci.* 1:4, 143-148.

Dahlstrom, M., Inasio, J., Jansson, E., Kaijser, L. (1996). Physical fitness and physical effort in dancers: a comparison of four major dance styles. *Impulse: Int. J. Dance Sci. Med. Ed.* 4:3, 193-209.

Galanti, M.L.A., Holland, G.J., Shafranski, P., Loy, S.F., Vincent, W.J., Heng, M.K. (1993). Physiological effects of training for a jazz dance performance. *J. Strength Cond. Res.* 7:4, 206-210.

Guidetti, L., Emerenziani, G., Gallotta, M., Baldari, C. (2007b). Effect of warm up on energy cost and energy sources of a ballet dance exercise. *Eur. J. Appl. Physiol.* 99:3, 275-281.

Guidetti, L., Emerenziani, G. P., Gallotta, M. C., Da Silva, S. G., Baldari, C. (2008). Energy cost and energy sources of a ballet dance exercise in female adolescents with different technical ability. *Eur. J. Appl .Physiol.* 103:3, 315-321. doi:10.1007/s00421-008-0705-y

Guidetti, L., Gallotta, M. C., Emerenziani, G. P., Baldari, C. (2007a). Exercise Intensities during a Ballet Lesson in Female Adolescents with Different Technical Ability. *Int. J. Sport Med.* 28:9, 736-742.

Kļonova, A., Kļonovs, J. (2010). Heart rate and energy consumption during standard sport dancing. *LASE J. Sport Sci.* 1:1, 48-52.

Koutedakis, Y., Hukam, H., Metsios, G., Nevill, A., Giakas, G., Jamurtas, A., Myszkewycz, L. (2007). The Effects of Three Months of Aerobic and Strength Training on Selected Performance and Fitness-Related Parameters in Modern Dance Students. *J. Strength Cond. Res.* 21:3, 808-812. doi:10.1519/R-20856.1.

Koutedakis, Y., Myszkewycz, L., Soulas, D., Papapostolou, V., Sullivan, I., & Sharp, N. C. (1999). The effects of rest and subsequent training on selected physiological parameters in professional female classical dancers. *Int. J. Sport. Med.* 20:6, 379-383. doi:10.1055/s-2007-971148

Liiv, H., Jurimae, T., Klonova, A., & Cicchella, A. (2013). Performance and recovery: stress profiles in professional ballroom dancers. *Med. Probl. Perform. Ar.* 28:2, 65-69..

Maciejczyk, M., & Feć, A. (2013). Evaluation of Aerobic Capacity and Energy Expenditure in Folk Dancers. *Hum. Movement.* 14:1, 76-81.

Martyn-Stevens, B.E., Brown, L.E., Beam, W.C., & Wiersma, L.D. (2012). Effects of a Dance Season on the Physiological Profile of Collegiate Female Modern Dancers. *Med. Sport.* 16:1, 1-5.

Massidda, M., Cugusi, L., Ibba, M., Tradori, I., Calò, C.M. (2011). Energy expenditure during competitive Latin American dancing simulation. *Med. Probl. Perform. Ar.* 26:4, 206–210.

Mistiaen, W., Roussel, N.A., Vissers, D., Daenen, L., Truijen, S., Nijs, J. (2012). Effects of aerobic endurance, muscle strength, and motor control exercise on physical fitness and musculoskeletal injury rate in preprofessional dancers: an uncontrolled trial. *J. Manip. Physiol. Ther.* 35:5, 381-389. doi:10.1016/j.jmpt.2012.04.014

Oliveira, S.M.L., Simões, H.G., Moreira, S.R., Lima, R. M., Almeida, J. A., Ribeiro, F. M. R., … Campbell, C. S. G. (2010). Physiological responses to a tap dance choreography: comparisons with graded exercise test and prescription recommendations. *J. Strength Cond. Res.*24:7, 1954-1959. doi:10.1519/JSC.0b013e3181ddae99

Ramel, E., Thorsson, O., Wollmer, P. (1997). Fitness training and its effect on musculoskeletal pain in professional ballet dancers. / Effets d' un entrainement pour ameliorer la condition physique sur les douleurs musculaires chez les danseurs de ballet professionnels. *Scand. J. Med. Sci. Spor.* 7:5, 293-298.

Redding, E., Weller, P., Ehrenberg, S., Irvine, S., Quin, E., Rafferty, S., … Cox, C. (2009). The Development of a High Intensity Dance Performance Fitness Test. *J. Dance Med. Sci.* 13:1, 3-9.

Rimmer, J.H., Jay, D., Plowman, S.A. (1994). Physiological characteristics of trained dancers and intensity level of ballet class and rehearsal. *Impulse: Int. J. Dance Sci. Med. Ed.* 2:2, 97-105.

Schantz, P.G., Astrand, P.O. (1984). Physiological characteristics of classical ballet. *Med. Sci. Sport. Exerc.* 16:5, 472-476.

Twitchett, E., Angioi, M., Koutedakis, Y., Wyon, M. (2009). Video analysis of classical ballet performance. *J. Dance Med. Sci.* 13:4, 124-128.

Wyon, M.A., Abt, G., Redding, E., Head, A., Sharp, N.C.C. (2004). Oxygen Uptake During Modern Dance Class, Rehearsal, and Performance. *J. Strength Cond. Res.*18:3, 646-649. doi:10.1519/13082.1

Wyon, M. A., & Redding, E. (2005). Physiological monitoring of cardiorespiratory adaptations during rehearsal and performance of contemporary dance. *J. Strength Cond. Res.* 19:3, 611-614. doi:10.1519/14233.1

Wyon, M. A., Twitchett, E., Angioi, M., Clarke, F., Metsios, G., & Koutedakis, Y. (2011). Time motion and video analysis of classical ballet and contemporary dance performance. *Int. J. Sports Med.* 32:11, 851-855. doi:10.1055/s-0031-1279718

Wyon, M., Head, A., Sharp, C., & Redding, E. (2002). The Cardiorespiratory Responses to Modern Dance Classes: Differences Between University, Graduate, and Professional Classes. *J. Dance Med. Sci.* 6:2, 41-45.
